# Polyphenol-Rich Extract of *Chrysanthemum* × *morifolium* (Ramat) Hemsl. (Hangbaiju) Prevents Obesity and Lipid Accumulation Through Restoring Intestinal Microecological Balance

**DOI:** 10.3390/plants14152393

**Published:** 2025-08-02

**Authors:** Xinyu Feng, Jing Huang, Lin Xiang, Fuyuan Zhang, Xinxin Wang, Anran Yan, Yani Pan, Ping Chen, Bizeng Mao, Qiang Chu

**Affiliations:** 1 Tea Research Institute, Zhejiang University, Hangzhou 310058, China; 12416099@zju.edu.cn (X.F.); 12416092@zju.edu.cn (L.X.); 3210102614@zju.edu.cn (F.Z.); 22316146@zju.edu.cn (X.W.); 12416100@zju.edu.cn (A.Y.); yanipan@zju.edu.cn (Y.P.); pingchen@zju.edu.cn (P.C.); 2 Institute of Landscape Architecture, Zhejiang University, Hangzhou 310058, China; 3 Institute of Biotechnology, Zhejiang University, Hangzhou 310058, China; 4 Ministry of Agriculture Key Lab of Molecular Biology of Crop Pathogens and Insects, Hangzhou 310058, China; 5 Zhejiang Key Laboratory of Biology and Ecological Regulation of Crop Pathogens and Insects, Hangzhou 310058, China

**Keywords:** Hangbaiju, polyphenols, obesity, hyperlipidemia, lipid accumulation, intestinal microbiota

## Abstract

*Chrysanthemum* × *morifolium* (Ramat) Hemsl. (Hangbaiju), which has been widely consumed as a herbal tea for over 3000 years, is renowned for its biosafety and diverse bioactivities. This study investigates the impact of polyphenol-rich Hangbaiju extracts (HE) on high-fat diet-induced obesity in mice. HE contains phenolic acids and flavonoids with anti-obesity properties, such as apigenin, luteolin-7-glucoside, apigenin-7-O-glucoside, kaempferol 3-(6″-acetylglucoside), etc. To establish the obesity model, mice were randomly assigned into four groups (*n* = 8 per group) and administered with either HE or water for 42 days under high-fat or low-fat dietary conditions. Administration of low (LH) and high (HH) doses of HE both significantly suppressed body weight growth (by 16.28% and 16.24%, respectively) and adipose tissue enlargement in obese mice. HE significantly improved the serum lipid profiles, mainly manifested as decreased levels of triglycerides (28.19% in LH and 19.59% in HH) and increased levels of high-density lipoprotein cholesterol (44.34% in LH and 54.88% in HH), and further attenuated liver lipid deposition. Furthermore, HE significantly decreased the *Firmicutes*/*Bacteroidetes* ratio 0.23-fold (LH) and 0.12-fold (HH), indicating an improvement in the microecological balance of the gut. HE administration also elevated the relative abundance of beneficial bacteria (e.g., *Allobaculum*, *norank_f__Muribaculaceae*), while suppressing harmful pathogenic proliferation (e.g., *Dubosiella*, *Romboutsia*). In conclusion, HE ameliorates obesity and hyperlipidemia through modulating lipid metabolism and restoring the balance of intestinal microecology, thus being promising for obesity therapy.

## 1. Introduction

In recent years, obesity has emerged as a prevalent social problem and chronic disease. Between 1980 and 2015, the global proportion of overweight individuals rose from 26.5% to 39.0%, while the obesity rate (BMI ≥ 30) surpassed 12.5% [1]. Hyperlipidemia, hypertension, type 2 diabetes, cardiovascular diseases, etc. are types of metabolic syndrome that always occur along with obesity and cause multiple organ dysfunction and increased mortality rates [2]. The prevalence of metabolic syndrome is increasing globally due to urbanization, sedentary lifestyles, dietary changes, etc. Genetic factors, insulin resistance, accumulation of dysfunctional adipose tissue and ectopic lipids, systemic inflammation, and dyslipidemia are perceived as contributing factors of metabolic syndrome [3]. Consequently, obesity has been defined as a “noncommunicable disease” [4]. Obesity and consequent metabolic syndrome have continuously aggravated the burden of health costs for both individuals and nations [5]. The pathogenesis of obesity is commonly identified as genetics, diet [6], and lifestyle [7] factors. The intestinal microbiome plays a critical role in maintaining host metabolic homeostasis and energy balance, thereby influencing the development and progression of obesity. Bacterial components such as lipopolysaccharides, as well as microbial metabolites, can affect lipid metabolism by modulating short-chain fatty acid production, altering bile acid utilization, and interacting with endocannabinoid-like signaling pathways and their receptors [8,9]. These findings highlight the gut microbiota as a promising therapeutic target for obesity prevention and treatment. Current treatments for obesity, such as bariatric surgery, pharmacotherapy and lifestyle change programs, shown varying degrees of effectiveness, yet are often accompanied by significant limitations and side effects. There are potential cardiovascular risks associated with commercial weight-loss medications, such as cardiac valve disorders induced by serotonin agonists and neuropathy caused by 2,4-dinitrophenol [10]. Moreover, weight loss is not sustainable in the long term for patients undergoing bariatric surgery [11]. As a result, the focus of recent research has switched to alternative dietary therapy. Studies have found that plants’ secondary metabolisms, abundant in flavonoids, polyphenols, and polysaccharides, have the potential to decrease total triglyceride (TG) levels, suppress inflammation-related cytokine expression, and stimulate the proliferation of beneficial gut microbiota [12,13,14].

Hangbaiju (referred to as the inflorescences of *Chrysanthemum* × *morifolium* (Ramat) Hemsl., Asteraceae) contains plenty of bioactive compounds and is known as the ‘One Root of Medicine and Food’ [15]. Hangbaiju, as a perennial Asteraceae plant, has a history of cultivation spanning four centuries, and was originally grown in Zhejiang Province, which is characterized by a subtropical monsoon climate [16]. The flowers of Hangbaiju are solitary or grow in clusters of several at the top of the stem and branches, characterized by white ray florets in several layers [17]. Its dried flowers have long been consumed as herbal tea. Hangbaiju is rich in bioactive compounds, including luteolin glycosides, chlorogenic acid, and dicaffeoylquinic acids, known for their anti-cancer, anti-mutagenic, anti-inflammatory, anti-oxidative, antidiabetic, and antihypertensive properties [15,18,19,20]. In a previous study, the content of caffeoylquinic acid ranged within 0.889 and 1.107 mg/g FW and that of flavonoids ranged within 47.425 and 49.376 mg/g FW, which accounted for 75.78–97.92% of total secondary metabolites [21]. Apigenin-7-O-glucoside, a common component of *Chrysanthemum* × *morifolium* (Ramat) Hemsl., exhibited inhibitory effects on the adipogenesis of 3T3-L1 cells and regulatory abilities to modulate gut microbiota [22,23]. Similarly, another common component, apigenin, was identified as a prebiotic for the regulation of gut composition in individuals with metabolic syndrome due to the sufficient time to interact with the intestine and gut microbiome caused by its low bioavailability [24]. Hangbaiju flavone could decrease the risk of hyperlipidemia through mediating the lipid metabolism and inhibiting the activity of enzymes [25]. Hangbaiju extract and its constituent luteolin both reduced lipotoxic intermediates, a key indicator of cardiovascular diseases, while exerting no regulatory effects on triacylglycerol [26]. Further research indicated that Hangbaiju extract can mitigate liver fat deposition and serum lipid levels (except high-density lipoprotein cholesterol, HDL-C) through downregulating the expression level of lipogenesis genes in epididymal adipose (EA) tissue [27]. Furthermore, several recent studies have further suggested the mitigation effects of Hangbaiju extract on gut microbiota disorder in metabolic dysfunction-associated fatty liver, and high-fat diet (HFD)-induced intestinal damage [28,29]. Yuan et al. discovered that *Chrysanthemum* water extract (200 mg kg^−1^) and high-dose treatment of its flavonoid components could significantly reduce the ratio of *Firmicutes*/*Bacteroidetes* (*F*/*B*), while a low dose of the flavonoids showed no significance. The probiotic *Akkermansia* was enriched by different doses of flavonoid treatment to different extents, but showed no evident increase in the Hangbaiju water extract-treated group [21]. Another study demonstrated that the water extract of Hangbaiju (4.2 g kg^−1^) elevated the abundance of *Akkermansia*, Rikenellaceae, and *Bacteroidales_RF16_group_unclassified* [22]. Despite these encouraging results, the comprehensive effects of major bioactive compounds in Hangbaiju on hepatic lipid deposition, dyslipidemia, and gut microbiota composition have not been systematically investigated.

This study aims to characterize the chemical profiles of Hangbaiju polyphenols and evaluate their effects on obesity-related parameters, hepatic lipid deposition and inflammation, and intestinal microbiota compositions in an HFD obesity mouse model. Fifteen phenolic compounds were identified from Hangbaiju extracts (HE). The regulatory effects of HE on obesity, liver metabolism, and intestinal microecology in HFD-treated obese mice were verified by histological evaluation, hematological examination, and 16S rDNA sequencing. However, details about the molecular mechanisms of anti-obesity action remain unexplored. This study shows the potential of HE to serve as an appropriate candidate for anti-obesity.

## 2. Results and Discussions

### 2.1. Characterization of HE Chemical Profiles

**Figure 1:**
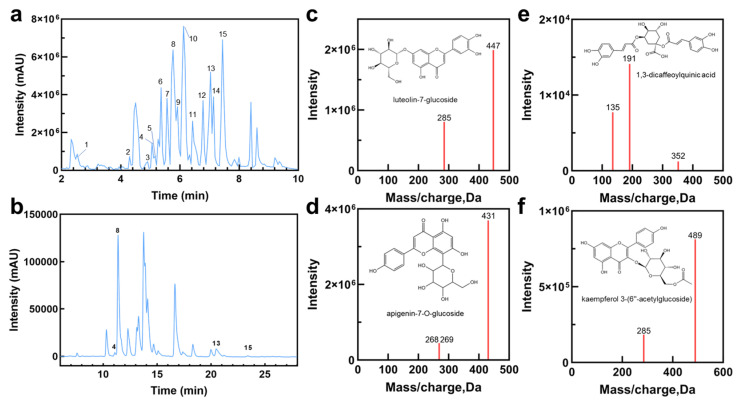
The chemical profiles of HE. (**a**) The base peak chromatogram of HE analyzed by UPLC–MS (1, caffeoyl hexoside; 2, benzyl-β-primeveroside; 3, Safflor yellow A; 4, 5,7,3′,5′-tetrahydroxyflavanone 7-O-glucuronide; 5, decaffeoyl verbascoside; 6, eriodicyol-7-O-glucoside; 7, 1,3-dicaffeoylquinic acid; 8, luteolin-7-glucoside; 9, 4,5-dicaffeoylquinic acid; 10, apigenin-7-O-glucoside; 11, okanin; 12, kaempferol 3-(6″-acetylglucoside; 13, luteolin; 14, apigenin-7-O-6″-acetyl-glucoside; 15, apigenin). (**b**) The HPLC chromatogram of flavonoids in HE. (**c**–**f**) The secondary mass spectrum of anti-obesity compounds in HE, including apigenin-7-O-glucoside 1,3-dicaffeoylquinic acid, luteolin-7-glucoside and kaempferol 3-(6″-acetyl glucoside).

HE was extracted using ultrasonics and purified by column chromatography for chemical chromatogram analysis. As indicated by the ultra-high-performance liquid chromatography–mass spectrometer (UPLC–MS) analysis results (Figure 1a, Table 1), the main chemical components of HE were phenolic acids, flavonoids, and flavonoid derivatives. Most flavonoids in Hangbaiju exist as glycosides, such as caffeoyl hexoside, benzyl-β-primeveroside, 5,7,3′,5′-tetrahydroxyflavanone 7-O-glucuronide, decaffeoyl verbascoside, apigenin-7-O-glucoside, luteolin-7-glucoside, eriodicyol-7-O-glucoside, kaempferol 3-(6″-acetylglucoside) and apigenin-7-O-6″-acetyl-glucoside. The main phenolic acids are phenylpropanoic acids with a C6-C1 structure, including 4,5-dicaffeoylquinic acid and 1,3-dicaffeoylquinic acid. The content of common flavonoids has been detected by high-performance liquid chromatography (HPLC) analysis (Figure 1b, Table S1). Apigenin-7-O-glucoside has been proved to inhibit adipogenesis in 3T3-L1 preadipocytes at early stages [23]. Previous research on *Chrysanthemum indicum* aqueous extract consisting of apigenin, kaempferol 3-(6″-acetyl glucoside), and 1,3-dicaffeoylquinic acid, which are also contained in HE (Figure 1c–f), has shown the inhibition of lipid droplet formation and alleviation of the expression of lipogenesis and adipogenesis-associated biomarkers [30]. It can be deduced that these compounds contributed to the potential bioactivities of HE.

### 2.2. HE Alleviates HFD-Induced Obesity and Hyperglycemia

**Figure 2:**
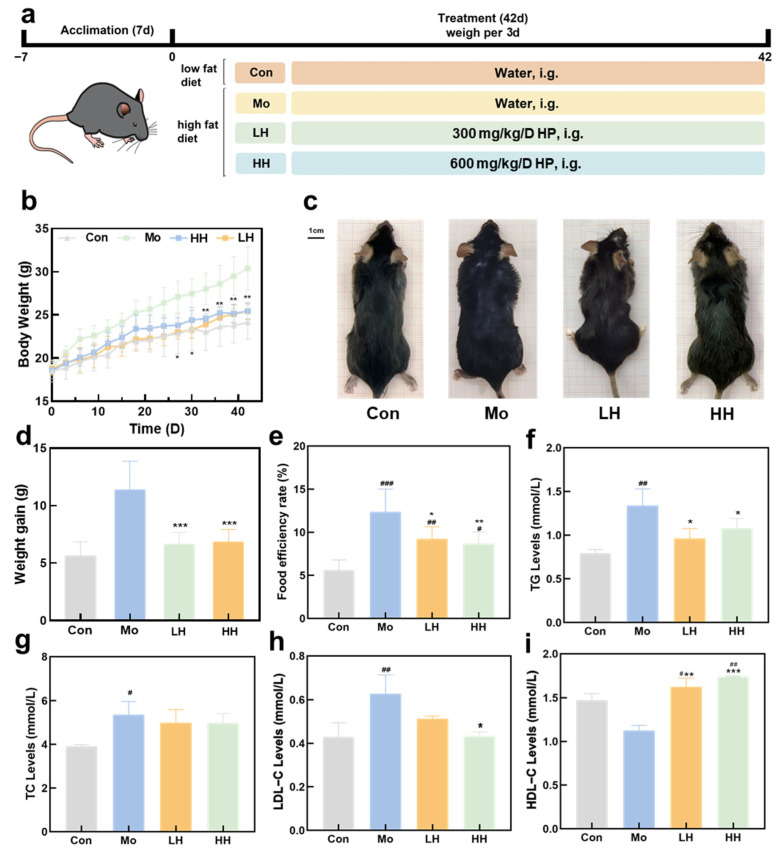
The ability of HE to regulate body weight, food intake, and serum adipose level. (**a**) Management scheme of animal experiments. (**b**) The body weight variations in the 4 different groups. (**c**) The body shape of mice after 42-day treatment. (**d**,**e**) The weight gain and food efficiency rate of different groups during the whole 42-day treatment. (**f**–**i**) The serum lipids levels (TG, TC, HDL-C, LDL-C) among four different groups. (*n* = 8, ^#^ *p* < 0.05, ^##^ *p* < 0.01, ^###^ *p* < 0.001, vs. the Con group; * *p* < 0.05, ** *p* < 0.01, *** *p* < 0.001, vs. the Mo group).

After 42 days of continuous HFD feeding, a mouse model of obesity was successfully established, followed by treatment with varying doses of HE (Figure 2a). From the 27th day, mice in the model (Mo) group exhibited a significantly heavier body weight compared to those in the other three groups (*p* < 0.05) (Figure 2b). Intuitively, after a 42-day intragastric administration of HE at 300 or 600 mg/kg per day, the body shape of mice in the low dose-treated HE (LH) and high dose-treated HE (HH) groups were more similar to those fed with a low-fat diet, while the body shape of the Mo group was much bigger than the other groups (Figure 2c), which preliminarily indicated the anti-obesity effect of HE. By the 42nd day, the weight gain of the LH and HH groups was maintained at a lower level when compared to the Mo group (*p* < 0.001) (Figure 2d). HE treatment exerted no significant effects on the daily food intake compared to the Mo group (Figure S1). Meanwhile, the daily food efficiency rate was calculated by the following formula: [increase of body weight (g)]/total food intake (g) × 100%]. The daily food efficiency rate of the LH and HH groups were significantly lower than that of the Mo group, indicating that HE treatment exerted a positive impact on the equilibrium between body weight and energy intake in HFD-treated mice. (Figure 2e) [37]. Serum lipid level indices also indicated that both low and high doses of HE could relieve hyperlipidemia. The TG level in mice from the LH and HH groups significantly decreased in comparison with that of the Mo group (Figure 2f). Although no significant differences were observed in total cholesterol (TC) levels, the HDL-C levels were notably increased following HE treatment. Low-density lipoprotein cholesterol (LDL-C) levels were significantly reduced in the HH group, while no notable change was observed in the LH group. Both the LH and HH groups showed higher HDL-C levels than the Mo group, even surpassing those in the control (Con) group (Figure 2h,i).

### 2.3. HE Ameliorates Adipose Tissue Enlargement and Attenuates Lipid Deposition and Inflammation in the Liver

**Figure 3:**
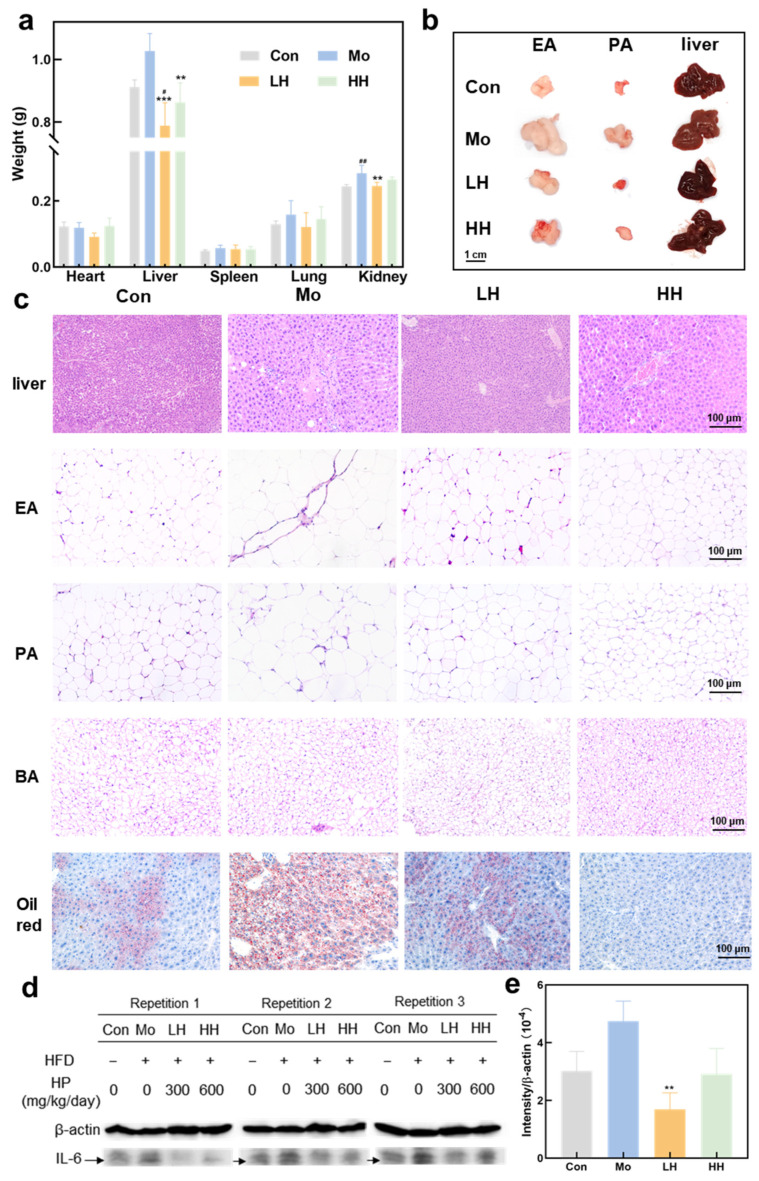
The improvement of HE on adipose tissue and lipid accumulation in the liver. (**a**) The weight of liver, heart, lung, kidney and spleen (*n* = 8, ^#^ *p*< 0.05, ^##^ *p* < 0.01, vs. the Con group; ** *p* < 0.01, *** *p* < 0.001, vs. the Mo group). (**b**) The images of EA, PA and liver. (**c**) The histopathology of liver, white and BA tissues stained with Oil Rred O and H&E. (**d**) Western blot analysis of IL-6 and β-actin. (**e**) Quantification of IL-6 band intensity (*n* = 3). Statistical significance: ** *p* < 0.01 vs. the Mo group.

Adipose tissue in mice is primarily distributed subcutaneously, around internal organs, and in the abdominal omentum. As shown in Figure 3a, compared to the Con group, organ weights (liver, spleen, lung, and kidney) were increased in the Mo group. Notably, the HE-treated groups exhibited a significant reduction in liver weight in contrast to the Mo group, while only the LH group demonstrated a remarkable decline in kidney weight. As the primary organ responsible for lipid processing, the liver gaining weight frequently occurs in parallel with obesity development [38]. The liver weight in HE-treated groups showed a significant decrease compared to the Mo group. Meanwhile, the livers of mice in the Mo group appeared visibly larger and more yellowish-brown, which is an indicator of ectopic fat deposition in the liver [39]. Furthermore, the EA and perirenal adipose (PA) tissues also showed a significant increase in volume (Figure 3b). These findings demonstrate the suppression effects of HE on weight gain and lipid accumulation in the liver in HFD-treated mice.

As shown in Figure 3c, liver histopathological analysis revealed that hepatocytes in the Con group were arranged in a uniform pattern. However, those in the Mo group exhibited irregular arrangement, accompanied by the existence of large lipid vacuolization, dense lipid droplets, and obvious inflammatory infiltration. HE treatment reversed the irregular arrangement of hepatocytes and abnormal lipid deposition in the liver. Adipocyte hypertrophy and an extension of the adipocyte area were observed in liver tissues of mice from the Mo group in comparison with those from the Con group. In contrast, adipocytes in the LH and HH groups exhibited a smaller size and were arranged more regularly. Furthermore, brown adipose (BA) is metabolically active and plays a role in preventing obesity and hyperlipidemia [40]. BA in the HE-treated groups presented as smaller in size and more regular in arrangement. It could be deduced that the HE-treated groups inhibited fat accumulation and stimulated white fat browning. Browning is known to enhance glucose and lipid metabolism and is considered as a promising strategy for obesity management [41].

Oil Red O staining was employed to further confirm lipid accumulation patterns. In line with the H&E staining results, Oil Red O staining also showed that liver tissues from the Mo group were characterized by the largest area of staining and the most intense color (Figure 3c). The accumulation of lipid droplets is considered to be a prominent feature of nonalcoholic fatty liver disease (NAFLD), which can progress to nonalcoholic steatohepatitis in response to inflammatory stimuli [42,43]. HE-treated groups significantly attenuated the pathological symptoms of liver deterioration. Hepatocytes displayed uniform size and radial arrangement with fewer lipid vacuoles, demonstrating the protective role of HE against hepatic steatosis and liver microstructural damage. Meanwhile, the area and depth of Oil Red O staining in all HE-treated groups showed different degrees of reduction, and high-dose treatment of HE showed more significant relief effects on lipid accumulation. The above results demonstrated that HE exerted a protective effect on liver microstructure and a preventive effect on the level of abnormal lipid accumulation, thereby restraining the further progression of hepatic steatosis.

Chronic inflammation is a common feature of obesity and contributes to elevated IL-6 and TNF-α levels, increasing the risk of liver diseases, including cancer [44]. The expression levels of IL-6 in the liver tissues were investigated through western blot analysis. Though there was no significant difference between the Mo group and the Con group, the average level of IL-6 in the Mo group was relatively higher, indicating the potential risk of development of liver inflammation and related diseases. As Figure 3d,e shows, a low dose of HE treatment significantly reduced the level of IL-6, demonstrating the prevention effects of HE on liver diseases. A high dose of HE treatment maintained the same level as the Con group and was lower than that of the Mo group, though it showed no significant difference with the Mo group. Reduction of hepatic IL-6 levels could ameliorate insulin resistance and hepatic lipid content in metabolic syndrome [45]. The results suggested the beneficial effects of HE on liver metabolism.

### 2.4. HE Altered Intestinal Microbiological Compositions of HFD Mice

**Figure 4:**
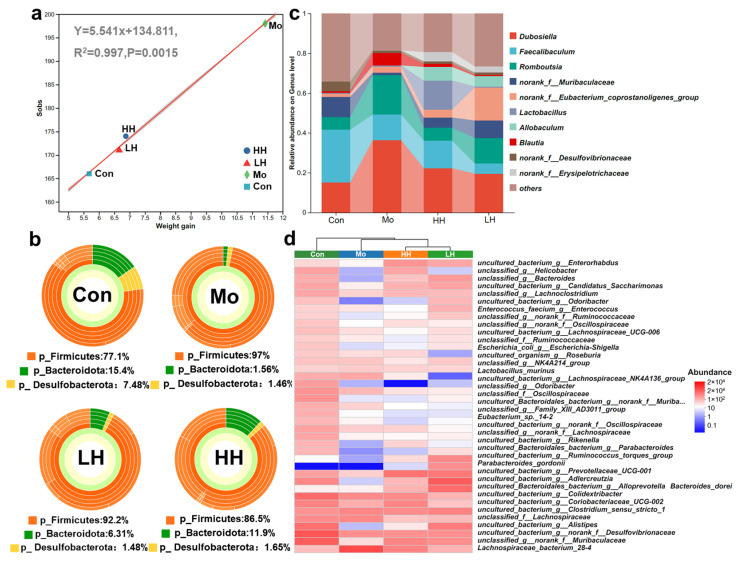
The changes in microbiota composition by HE. (**a**) The regression curve between the Sobs index at the OTU level and weight gain in different groups. (**b**) The sunburst diagram and the intestinal microbiological compositions at the phylum level among 4 different groups. (**c**) The intestinal microbiota community bar plot analysis at the genus level. (**d**) The heatmap diagram of communities at the species level.

Environmental factors such as diet, genetics, sex, and pathological conditions collectively influence the composition of gut microbiota [46]. Previous studies have shown that *Chrysanthemum* water extract can mitigate intestinal microbiota dysbiosis. However, the modulation effects of phenolic compounds, the most essential constituent, have not been explored [28,29]. The α diversity of the intestinal microbiota community in different treatment groups was assessed. In this study, the Sobs index of the mice in the Mo group (401) surpassed markedly that in the Con group (260), reflecting the altered α diversity induced by HFD treatment [47]. The Sobs index of the two HE-treated groups was close to that of the Con group (309 in LH and 305 in HH), in line with the low level of body weight gain (Figure 4a). Consistently, the Shannon index of the Mo group also increased 1.755-fold compared to the Con group, and HE treatment reversed the alteration, decreasing 0.847- and 0.875-fold in the LH and HH groups compared to the Mo group, respectively. A regression analysis revealed a strong correlation between the Sobs index and weight gain (R^2^ = 0.997, *p* = 0.0015), suggesting a close interaction between gut microbiota and obesity. To explore the structural composition of the microbiota, the relative abundance of dominant bacteria at different taxonomic levels was statistically analyzed. At the phylum level (Figure 4b), *Firmicutes* and *Bacteroidetes* were the predominant phyla. HFD feeding resulted in a significant increase in *Firmicutes* while causing a remarkable decrease in *Bacteroidetes*. The *F*/*B* ratio serves as an indicator of intestinal microecological balance and is widely viewed as a typical parameter to assess the health of the gut microbiota [48]. In clinical studies, *Bacteroidetes* were inversely associated with body fatness and waist circumference, whereas *Firmicutes* showed a positive correlation with body fat [48]. The *F*/*B* ratio of the Mo group displayed a 12.44-fold increase compared to the Con group. HE intervention, especially the high-dose treatment of HE, significantly decreased the *F*/*B* ratio 0.23-fold (LH group) and 0.12-fold (HH group) compared to the Mo group. The decrease of *F*/*B* indicated the prevention effects of HE on gut microbiota dysbiosis and the potential effects on the inhibition of obesity development.

At the genus level (Figure 4c), the investigation of community abundance showed that the predominant microbiota consisted of *Dubosiella*, *Faecalibaculum*, *Romboutsia*, *norank_f__Muribaculaceae*, *norank_f__Eubacterium_coprostanoligenes_group*, *Lactobacillus*, *Allobaculum*, *Blautia*, etc. The abundance of *Dubosiella* (2.407-fold increase), *Romboutsia* (3.114-fold increase), and *Blautia* (7.147-fold increase) in the Mo group was significantly higher than in the Con groups, which are enriched in obese individuals and grow in sync with the progression of obesity and fat accumulation [49]. In comparison, there was an obvious decline in the abundance of *norank_f__Muribaculaceae* (0.125-fold decrease) and *Faecalibaculum* (0.485-fold decrease) in the Mo group. The HE treatment reversed the alteration of *Blautia*, *Romboutsia*, *Dubosiella*, and *norank_f__Muribaculaceae*. In addition, low and high doses of HE treatment elevated the relative abundance of *Allobaculum* (5.26% and 6.87%) and *norank_f__Erysipelotrichaceae* (3.03% and 4.66%) compared to the Mo (0.08%) and Con (0.02%) groups. *Allobaculum*, a short-chain fatty acid producer, has been proven to be resistant to the development of NAFLD and is also attributed to the reduction of body weight in obese mice [50]. *Norank_f__Muribaculaceae* and *norank_f__Erysipelotrichaceae*, which negatively correlated with obesity-associated indices, were supposed to play a critical role in attenuating abnormal lipid metabolism and suppressing obesity development [51,52]. Furthermore, the abundance of *Bacteroides dorei* and *Parabacteroides gordonii* was tremendously elevated by HE treatment, while that of *unclassified_f_Oscillospiraceae* (0.38-fold and 0.308-fold decrease in the HH and LH groups) and *uncultured_bacterium_g_Lachnospiraceae_NK4A136_group* (0.203-fold and 0.003-fold decrease in HH and LH groups) reduced in both the HH and LH groups (Figure 4d). Oral administration of *Bacteroides dorei* has been proven to enhance BA catabolism and inhibit body weight growth [53]. *P. gordonii* showed a significant inverse correlation between body mass index, while an increased abundance of *uncultured_bacterium_g_Lachnospiraceae_NK4A136_group* and *unclassified_f__Oscillospiraceae* was commonly found in the HFD-induced obesity model [54,55,56]. These findings suggest that HE treatment modulates gut microbiota composition in a manner beneficial for obesity prevention.

### 2.5. HE Treatment Intervened in the Serum Lipid Profiles and Weight Growth Through Regulating Intestinal Microbiota

**Figure 5:**
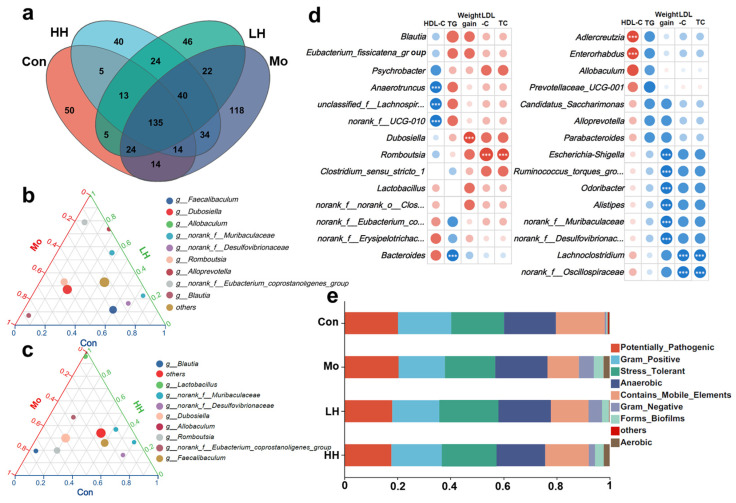
HE alleviated obesity-induced hyperlipidemia through gut microbiota modulation. (**a**) The Venn diagram depicting the distribution of OTUs among different groups. (**b**,**c**) The quasi-ternary phase chart illustrating the gut microbiota composition at the species level for the four different groups. (**d**) The heatmap of the relationship between obesity indices and bacteria communities abundance (*** *p* < 0.001). (**e**) The BugBase phenotype prediction of bacterial community in different groups.

HE treatment vastly altered the community composition of gut microbiota, whether compared to the Con or the Mo group. According to the Venn diagram (Figure 5a), 109 of the total 265 taxonomic units (OTUs) were common to all samples. The Mo group had 118 unique OTUs, while the LH and HH groups had 46 and 40 unique OTUs, respectively, indicating distinct microbiota profiles. The ternary plots (Figure 5b,c) further illustrated the different gut microbiological compositions at the species level. The gut flora of the LH group was characterized by *g_Allobaculum*, *g_norank_f_Eubacterium_coprostanoligenes_group* and *g_Alloprevotella*, whereas the HH group was characterized by *g_Allobaculum* and *g_Lactobacillus*. Previous studies have shown that oral luteolin increases *Allobaculum* abundance, which is negatively correlated with lipopolysaccharide levels and pathogenic bacteria [57]. A previous study also found that 0.5 mg/mL *Chrysanthemum morifolium* flower extract (containing luteolin, apigenin, apigenin-7-O-glucoside, etc.) exerted no effects on the proliferation of *Lactobacillus* in vitro, while 1.0–4.0 mg/mL extract significantly boosted the growth of *Lactobacillus* sp. [58]. It can be speculated that the dose–effect relationship resulted in the different regulatory effects of low and high doses of HE administration on the intestinal microbiological compositions in HFD-treated mice. Correlation analysis between microbiota and obesity-related indices (Figure 5d) revealed that *Dubosiella* was significantly positively associated with weight gain, and *Romboutsia* correlated with serum TC and LDL-C levels. *Dubosiella* has been found to be enriched in *Lcn2*-knockout mice under HFD-fed conditions, playing an important role in obesity through regulating microbial metabolites [59]. *Romboutsia* is also positively associated with serum lipids, uric acid, and weight gain in clinical cohorts [60]. *Dubosiella* and *Romboutsia* have been identified as the dominant bacteria in the Mo group, indicating their contributions to the development of obesity. In contrast, *unclassified_f__Lachnospiraceae*, *norank_f_UCG-010* and *Anaerotruncus* were negatively associated with HDL-C levels. Meanwhile, *norank_f_Muribaculaceae*, *Odoribacter*, and *Alistipes* showed distinct negative correlations with weight gain, and *Lachnoclostridium* and *norank_f_Oscillospiraceae* exhibited a significant inverse association with the levels of TC and LDL-C. To further assess the physiological implications of HE-induced microbiota changes, microbial phenotype predictions were performed using BugBase (Figure 5e). HE treatment reduced the abundance of potentially pathogenic bacteria and Gram-negative species. In contrast, phenotypes associated with mobile elements and stress tolerance were enriched in the HE-treated groups compared to the Mo group, suggesting enhanced microbial resilience and reduced pathogenicity.

## 3. Materials and Methods

### 3.1. Materials

Hangbaiju samples were cultivated at the breeding demonstration base in the Agricultural-Technology Extension and Service Center (Tongxing, Zhejiang, China) and harvested in late October during the peak flowering period [61,62]. The Hangbaiju flowers were firstly steamed using a steamer at 130 °C, and then spread out for 2 h to cool. Then all Hangbaiju samples were placed into a 60 °C blast oven for 8–10 h to dry and acquire final products. The standard samples for chromatographic analysis, including luteolin, luteolin-7-glucoside, apigenin, and myricitrin, were obtained from Yuanye Bio-Technology Co., Ltd. (Shanghai, China). Chemical reagents (formic acid, ethanol, Oil Red O, phosphate buffered saline and acetonitrile) were obtained from Solarbio Technology Co., Ltd. (Beijing, China).

### 3.2. Preparation and Purification of HE

Samples totaling 2 kg of dried Hangbaiju were crushed and immersed in 80% ethanol in a 1:5 (*w*:*v*) ratio. Then ultrasonic extraction (40 kHz, 100 W) was performed at 45 °C for 90 min. After filtration and sedimentation at 4 °C overnight, the samples were centrifuged for 10 min at 10,000× *g* and the supernatant was vacuum concentrated to 100 mL at 50 °C. After overnight sedimentation, the samples underwent additional centrifugation at 9500× *g* for 10 min. The collected supernatant was then loaded onto a D101 macroporous resin column (3.6 × 60 cm) and eluted with distilled water (2× column volume) and 80% ethanol (1× column volume). The eluate was collected, condensed through rotary distillation, and lastly vacuum freeze-dried and stored at −80 °C. The final production yield of HE was 0.90%.

### 3.3. The Characterization of HE Chemical Profiles

#### 3.3.1. UPLC–MS Analysis

The chemical constituents of HE were identified using the HPLC system and UPLC–MS system. Firstly, HE was dissolved in ultrapure water and filtered through a 0.22 µm aqueous membrane for subsequent analysis. For the UPLC–MS analysis, chromatographic separation was performed using a BEH C18 column (2.1 × 100 mm, 1.7 μm, Waters^®^, Milford, CT, USA) with a flow rate of 0.3 mL/min. The mobile phases A were prepared with 0.1% formic acid in ultrapure water, and the mobile phases B were acetonitrile. The gradient elution procedure was performed as follows: 0–3 min, 20% B; 10–12 min, 100% B; 15–19 min, 95% B; 20–21 min, 5% B. Mass spectrometry was performed under a negative ionization mode using an electrospray ionization (ESI) source, with data acquired in full-scan mode. The electrospray ionization voltage was set at 3.2 kV, and the flow rate and temperature of the sheath gas were set at 12 L/min and 350 °C. The acquired mass scan ranged from 50–1000 (*m*/*z*).

#### 3.3.2. Identification of UPLC–MS Analysis

The identification of the phenolic constituents was conducted with the Agilent MassHunter software (version 7.0), where the molecular formula of the test substance can be calculated and the *m*/*z*, secondary mass spectrometry, and retention time of the chemical composition represented by each chromatographic peak can be acquired with a small margin of error. The Reaxy website (https://www.reaxys.com/, accessed on 22 February 2025), NIST Mass Search 2.3, the MassBank database, and the NIST 17 database were used for identification. Once the molecular weight, speculated formula, and mass error of compounds were acquired from the software, the MS/MS spectra were introduced to NIST Mass Search 2.3. If the compounds were not concluded in NIST 17, then the speculated formula was searched on the Reaxy website and MassBank and compared to related references.

#### 3.3.3. HPLC Analysis

The HPLC analysis method was followed and modified based on our previous study [63]. Chromatographic separation was performed using a Shimadzu LC-20 A HPLC system (Shimadzu Corporation, Kyoto, Japan) with an Agilent TC-C18 column (4.6 mm × 250 mm). The mobile phase A was formic acid solution in ultrapure water at a 1:999 ratio (*v*:*v*), while the mobile phase B contained formic acid and acetonitrile in a 1:999 proportion (*v*:*v*). Chromatographic separation was carried out at a flow rate of 1 mL/min with the column temperature maintained at 25 °C, adopting a 10 μL injection volume. The wavelength for the ultraviolet detector was performed at 360 nm.

### 3.4. Experimental Management of Animals

Male C57BL/6 mice (6 weeks of age) were purchased from Shanghai Slac Laboratory Animal Co., Ltd. (Shanghai, China). All the animal experiments were carried out according to the ethical requirements of the Experimental Animal Welfare Ethics Committee of Zhejiang University. The mice were first acclimated to the laboratory for 1 week in the animal care facility with the temperature maintained at 22 °C and 50% relative humidity, and underwent a 12/12 h light/dark cycle. Then, all the mice were divided into four groups (*n* = 8), namely the Con group, the Mo group, the LH group, and the HH group. All mice except those in the Con group were placed on a free HFD supply (ingredients and nutrition facts are listed in Tables S2 and S3). The LH and HH groups received daily intragastric gavage of HE at doses of 300 mg/kg per day and 600 mg/kg per day, respectively. The food consumption and body mass of each mouse were recorded every 2 days. Following 42 days of treatment, the mice were subjected to a 12-h fast and then sacrificed. Blood samples were acquired from bulbus oculi vessels, and the feces, livers, EA and PA tissues were harvested, weighed, and stored at −80 °C for subsequent analysis.

### 3.5. Histological Analysis

The livers, PA, EA and BA were cut and immersed in 4% paraformaldehyde solution for fixation over a 24-h period. 6-μm-thick tissue sections were prepared following gradient dehydration and paraffin embedding and were stained with hematoxylin and eosin (H&E). Then, Oil Red O staining was conducted on the sections of liver tissues, which were rehydrated and washed with phosphate buffered saline containing Tween 20. Microscopic observation of the tissue sections was conducted using a Zeiss microscope (Oberkochen, Germany).

### 3.6. Biochemical Measurement

Blood samples were centrifuged at 1500× *g* for 10 min at 4 °C to collect the serum. The concentrations of TC, TG, HDL-C and LDL-C were determined using enzymatic colorimetric assays with a reagent kit (Nanjing Jiancheng Bioengineering Institute, Nanjing, China).

### 3.7. Western Blot Analysis

The liver tissues were crushed and dissolved in 50 mg of tissue/mL protein lysis buffer and incubated for 30 min. After centrifugation the supernatant was transferred to new tubes. After the determination of protein content, the homogenate proteins were loaded onto gel for separation. Then proteins were transferred to membranes and blocked for 2 h. The primary antibodies against IL-6 and β-actin were incubated with the membrane. ECL reagents were used for exposure and chemiluminescence, and a multifunctional imaging system (Shenhua, Hangzhou, China) was used for detection.

### 3.8. Gut Microbiota Assay

The genomic DNA of fecal samples was extracted using a QiAamp DNA stool Mini Kit (Qiagen, Hilden, Germany) and amplified with the primers 338F and 806R (fwd 5′-ACTCCTACGGGAGGCAGCAG-3′, rev 5′-GGACTACHVGGTWTCTAAT-3′) of the 3–4 variable region in the 16S rRNA gene sequence. Then the amplified samples underwent sequencing and bioinformatics analysis on the Illumina MiSeq platform, and were then analyzed by the Majorbio Cloud Platform (https://www.majorbio.com, accessed on 7 March 2025).

## 4. Conclusions

In this study, the chemical profiles of HE and the potential preventive effects against obesity and abnormal serum lipid levels were characterized. The phenolic compounds of HE are mainly composed of two phenolic acids (1,3-dicaffeoylquinic acid and 4,5-dicaffeoylquinic acid) and various flavonoids (e.g., luteolin-7-glucoside, apigenin-7-O-glucoside, kaempferol 3-(6″-acetylglucoside)). HE effectively restrained the growth of body weight and liver weight gain, as well as adipose tissue enlargement, in HFD-treated obese mice. The anti-hyperlipidemia abilities of HE are mainly reflected in the modulating effects on serum levels of TG and HDL-C, thereby reversing the abnormalities in lipid metabolism. Histological analysis showed that HE ameliorated adipose tissue enlargement and attenuated lipid accumulation in liver tissues. In addition, the HE-treated groups reconstructed the composition of gut microbes, resulting in a decrease in *Dubosiella* and *Romboutsia*, while stimulating a significant increase in *Allobaculum*, *norank_f__Erysipelotrichaceae*, and *norank_f__Muribaculaceae*. Our results suggested that HE alleviates obesity and hyperlipidemia by improving adipose tissue structure and altering intestinal microbiota composition.

## Figures and Tables

**Table 1:** Identification of HE components.

No.	*Rt* (min)	Observed *m*/*z*	Formula	Mass Error (ppm)	MS/MS	Tentative Identification	Category	Reference
1	2.527	341.0886	C_15_H_18_O_9_	−2.04	89, 135, 161, 179, 207, 251	Caffeoyl hexoside	Flavonoid glycoside	[31]
2	4.299	401.1457	C_18_H_26_O_10_	−1.25	101, 161, 233, 269	Benzyl-β-primeveroside	Flavonoid glycosides	[32]
3	4.906	593.1518	C_27_H_30_O_15_	−0.64	353, 383, 473	Safflor yellow A	Flavonoid	NIST 17
4	5.075	463.0892	C_21_H_17_O_12_	−2.15	113, 151, 175, 287	5,7,3′,5′-Tetrahydroxyflavanone 7-O-glucuronide	Flavonoid glycosides	[33]
5	5.176	461.1668	C_20_H_30_O_12_	−0.84	59, 89, 149, 191, 287, 415	Decaffeoyl verbascoside	Flavonoid glycosides	[34]
6	5.362	449.1111	C_21_H_22_O_11_	−4.1	135, 151, 287	Eriodicyol-7-O-glucoside	Flavonoid glycosides	NIST 17
7	5.564	515.1214	C_25_H_24_O_12_	2.9	135, 191, 352	1,3-Dicaffeoylquinic acid (cynarin)	Caffeoyl quinic acid derivatives	NIST 17 [35]
8	5.75	447.098	C_21_H_20_O_11_	−2	285, 447	Luteolin-7-glucoside	Flavonoid glycosides	[33]
9	5.936	515.1215	C_25_H_24_O_12_	−3.36	173, 353, 515	4,5-Dicaffeoylquinic acid	Caffeoyl quinic acid derivatives	[33]
10	6.138	431.1049	C_21_H_20_O_10_	−0.9	268, 269, 311	Apigenin-7-O-glucoside	Flavonoids and their glycosides	[33]
11	6.425	287.0569	C_15_H_12_O_6_	−2.53	65, 107, 135, 151	Okanin	Flavonoids derivatives	[33]
12	6.78	489.1054	C_23_H_22_O_12_	−2.55	285, 489	Kaempferol 3-O-β-D-6″-acetylglucoside	Flavonoid glycosides	[36]
13	6.999	285.0427	C_15_H_10_O_6_	−6.7	65, 107, 133, 175	Luteolin	Flavonoid glycosides	[33]
14	7.134	473.1105	C_23_H_22_O_11_	−2.54	63, 151, 240, 268, 311	Apigenin-7-O-6″-acetyl-glucoside	Flavonoid glycosides	[33]
15	7.438	269.0492	C_15_H_10_O_5_	−3	107, 117, 151, 201, 225	Apigenin	Flavonoid	NIST 17

## Data Availability

The data supporting this article have been included as part of the Supplementary Information.

## Supplementary Materials

The following supporting information can be downloaded at: https://www.mdpi.com/article/10.3390/plants14152393/s1, Figure S1: Daily food intake of different groups of mouse per day; Table S1: The content of common flavonoids and derivatives contained in HE; Table S2: The ingredients and nutrition facts of low-fat diets; Table S3: The ingredients and nutrition facts of high-fat diets.
