# Supplementary material for: Polyphenol-Rich Extract of Chrysanthemum × morifolium (Ramat) Hemsl. (Hangbaiju) Prevents Obesity and Lipid Accumulation Through Restoring Intestinal Microecological Balance
Source: Plants (Basel). 2025 Aug 2;14(15):2393. doi: 10.3390/plants14152393 (PMC12349671; doi:10.3390/plants14152393)
Supplement: Supplementary file 1 [file plants-14-02393-s001.zip › plants-3778555-supplementary.pdf]

Table S1 The ingredients and nutrition facts of high-fat diets and low-fat diets

D12450J	gm%	kcal%
Protein	19.2	20.0
Carbohydrate	67.3	70.0
Fat	4.3	10.0
Total		100
Kcal/gm	3.85	
Ingredient		
Casein, 80 Mesh	200	800
L-Cystine	3	12
Corn Starch	506.2	2024.8
Maltodextrin 10	125	500
Sucrose	68.8	275.2
Cellulose, BW200	50	0
Soybean Oil	25	225
Lard	20	180
Mineral Mix S10026	10	0
DiCalcium Phosphate	13	0
Calcium Carbonate	5.5	0
Potassium Citrate, 1 H20	16.5	0
Vitamin Mix V10001	10	40
Choline Bitartrate	2	0
FD&C Yellow Dye #5	0.04	0
FD&C Red Dye #5	0	0
FD&C Blue Dye #1	0.01	0
Total	1055.05	4057

Table S2 The ingredients and nutrition facts of high-fat diets and low-fat diets

D12492	gm%	kcal%
Protein	26.2	20
Carbohydrate	26.3	20
Fat	34.9	60
Total		100
Kcal/gm	5.24	
Ingredient		
Casein, 80 Mesh	200	800
L-Cystine	3	12
Corn Starch	0	0
Maltodextrin 10	125	500
Sucrose	68.8	275.2
Cellulose, BW200	50	0
Soybean Oil	25	225
Lard	245	2205
Mineral Mix S10026	10	0
DiCalcium Phosphate	13	0
Calcium Carbonate	5.5	0
Potassium Citrate, 1 H20	16.5	0
Vitamin Mix V10001	10	40
Choline Bitartrate	2	0
FD&C Yellow Dye #5	0	0
FD&C Red Dye #5	0.05	0
FD&C Blue Dye #1	0	0
Total	773.85	4057

Table S3 Identification of HP

No.	*Rt* (min)	observed *m/z*	formula	mass error (ppm)	MS/MS	tentative identification	Category
1	2.527	341.0886	$C_{15}H_{18}O_9$	-2.04	89, 135, 179, 207, 251	Caffeoyl hexoside	Flavonoid glycoside
2	4.299	401.1457	$C_{18}H_{26}O_{10}$	-1.25	101, 161, 233, 269	Benzyl-β-primeveroside	Flavonoid glycosides
3	4.906	593.1518	$C_{27}H_{30}O_{15}$	-0.64	353, 383, 473	Safflor yellow A	Flavonoid
4	5.075	463.0892	$C_{21}H_{20}O_{12}$	-2.15	113,151,175,287	Myricitrin	Flavonoid glycosides
5	5.176	461.1668	$C_{20}H_{30}O_{12}$	-0.84	59, 89, 149, 191, 287, 415	Decaffeoyl verbascoside	Flavonoid glycosides
6	5.362	449.1111	$C_{21}H_{22}O_{11}$	-4.1	135,151	Eriodicyol-7-O-glucoside	Flavonoid glycosides
7	5.564	515.1214	$C_{25}H_{24}O_{12}$	2.9	135, 191, 352	1,3-Dicaffeoylquinic acid	Caffeoyl quinic acid derivatives
8	5.75	447.098	$C_{21}H_{20}O_{11}$	-2	285,447	Luteolin-7-glucoside	Flavonoid glycosides
9	5.936	515.1215	$C_{25}H_{24}O_{12}$	-3.36	173,353,515	4,5-Dicaffeoylquinic acid	Caffeoyl quinic acid derivatives
10	6.138	431.1049	$C_{21}H_{20}O_{10}$	-0.9	269, 311	Apigenin-7-O-glucoside	Flavonoids and their glycosides
11	6.425	287.0569	$C_{15}H_{12}O_6$	-2.53	65, 107, 135,151	2',3,4,4',6'-Pentahydroxychalcone	Flavonoids derivatives
12	6.78	489.1054	$C_{23}H_{22}O_{12}$	-2.55	285,489	Kaempferol 3-O-β-D-6''-acetylglucoside	Flavonoid glycosides
13	6.999	285.0427	$C_{15}H_{10}O_6$	-6.7	65, 107, 133, 175	Luteolin	Flavonoid glycosides
14	7.134	473.1105	$C_{23}H_{22}O_{11}$	-2.54	63, 151, 240, 268, 311	Apigenin-7-O-6''-acetyl-glucoside	Flavonoid glycosides
15	7.438	269.0492	$C_{15}H_{10}O_5$	-3	107,117,151,201,225	Apigenin	Flavonoid

Table S4 The content of common flavonoids and derivatives contained in HP

No.	Compounds name	Retention time (min)	Content (mg/g)
**4**	Myricitrin	11.10	4.730 ± 0.004
**8**	Luteolin-7-glucoside	11.34	53.220 ± 0.004
**13**	Luteolin	20.50	12.693 ± 0.135
**15**	Apigenin	23.50	1.211 ± 0.141
